# PAK5‐stabilized Smuc confers renal cell carcinoma metastasis

**DOI:** 10.1002/ctm2.559

**Published:** 2021-09-16

**Authors:** Fu‐Chun Huo, Zhi‐Man Zhu, Qiu‐Ying Du, Dong‐Sheng Pei

**Affiliations:** ^1^ Department of Pathology Xuzhou Medical University Xuzhou Jiangsu China; ^2^ Department of Basic Medicine Jiangsu College of Nursing Huai'an China

Dear Editor,

Abundant evidence has demonstrated that PAK5 confers an oncogenic potential in carcinogenesis and tumor progression. Our previous discoveries have confirmed that PAK5 promotes the growth of breast cancer and metastasis of cervical cancer respectively through phosphorylating NF‐κB‐p65 and SATB1.^1^, ^2^ And microRNA‐106‐5p targets PAK5 to inhibit cell migration and invasion in RCC.^3^ However, the mechanism by which RCC acquires malignant phenotypes conferred by PAK5 remains unclear.

Snail and Slug are extremely unstable proteins, which can be explained by posttranslational modification.^4^ We speculated that whether Smuc possesses the potential characteristics similar to the other two members, and there is a previously unknown direct linkage between serine/threonine PAK5 kinase and Smuc. Western blot was performed to confirm the overexpression of PAK5 and Smuc (Figure S1A,B). Notably, reciprocal immunoprecipitation and co‐localization of immunofluorescence staining indicated the physical interaction between PAK5 and Smuc (Figure 1A; Figure S1C,D). We calculated and predicted four putative PAK5 phosphorylation sites (Ser63, Ser98, Ser125, Ser278) of Smuc according to the consensus PAKs phosphorylation residues,^5^ whereas only the motif surrounding Ser278 is evolutionarily conserved across other species (Figure 1B). We performed the in vitro phosphorylation assay using recombinant PAK5 proteins and various synthetic peptides containing unphosphorylated or phosphorylated residues of Smuc. PAK5 promoted the phosphorylation of the peptide containing the potential PAK5‐phosphorylation site (Ser278) of Smuc (Figure 1C). However, phosphorylation level of Smuc was effectively inhibited by the addition of homolog peptides harboring the random disruption of Smuc sequences or single‐site mutation of Ser278 replaced by alanine(Figure 1C).

**FIGURE 1:**
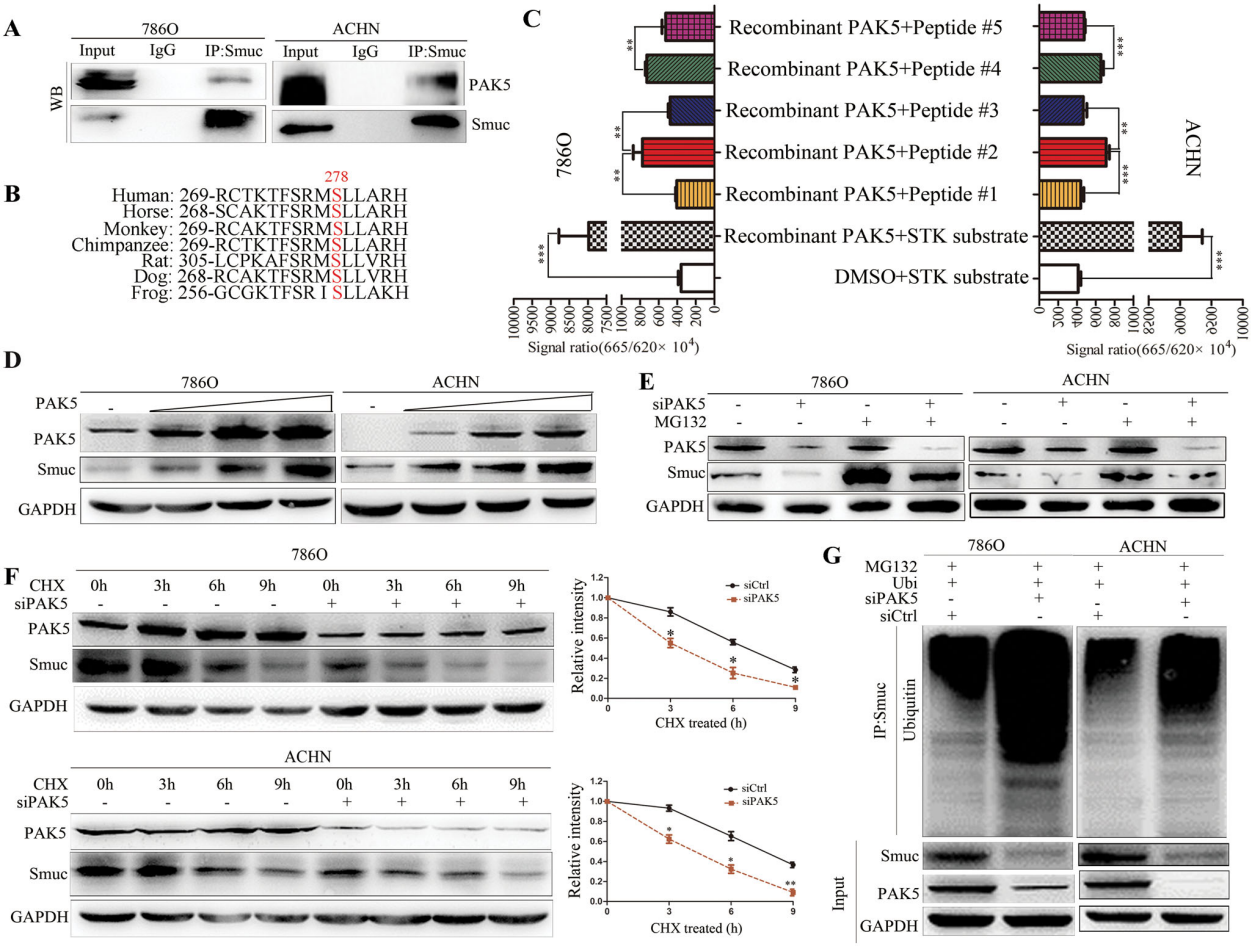
PAK5 phosphorylates and stabilizes Smuc. (A) Exogenous interaction between PAK5 and Smuc was determined by immunoprecipitation with the anti‐Smuc antibody in RCC cells. Immunoglobulin (Ig) G serves as negative control. (B) The motif surrounding Ser278 of Smuc is evolutionarily conserved across other species. (C) In vitro phosphorylation assay was performed in RCC cells using recombinant PAK5 proteins and various synthetic peptides (peptide #1: random disruption of the potential PAK5‐phosphorylation site/Ser278 of Smuc; peptide #2: potential PAK5‐phosphorylation site/Ser287 of Smuc; peptide #3: single‐site mutation of Ser278 to alanine of Smuc; peptide #4 [positive control]: identified PAK5‐phosphorylation site/Ser39 of E47; peptide #5: single‐site mutation of Ser39 to alanine of E47). E47 that has been reported to be phosphorylated at Ser39 by PAK5 in colon cancer was adopted as a positive control. (D) After transfecting PAK5 in a dose‐dependent manner, the expression of Smuc and PAK5 in protein levels was examined by western blot. (E) PAK5 interference decreased the protein expression of Smuc in RCC cells, which was blocked by the proteasome inhibitor MG132. (F) After the treatment with cycloheximide (CHX, a protein synthesis inhibitor) in RCC cells, lysates were collected at the indicated time and endogenous Smuc expression was tested by immunoblot. (G) After the indicated transfection, cells were treated with MG132. The ubiquitinated Smuc was detected. *p < 0.05; **p < 0.01; ***p < 0.001

Snail and Slug are short half‐life proteins targeted by the ubiquitin‐proteasome system.^6^ It has been reported that PAK1‐mediated phosphorylation of Snail regulates its subcellular localization and functions.^7^ Addition of Smuc protein rather than mRNA levels were more pronounced following the ectopic expression of PAK5 (Figure 1D; Figure S1E,G). Conversely, PAK5‐KO significantly inhibited Smuc expression (Figure S1F). Smuc expression was increased after MG132 treatment in RCC cells silencing PAK5 (Figure 1E). Cycloheximide pulse‐chase assays demonstrated that blockade of PAK5 could impair the half‐life of Smuc protein (Figure 1F). And silencing PAK5 elevated the ubiquitination levels of Smuc protein (Figure 1G). Taken together, PAK5 enhances Smuc stability through impairing ubiquitination‐dependent Smuc degradation.

The phosphorylation status of Snail acts a crucial role in regulating its stability.^8^ Herein, we explored the relationship between PAK5‐mediated Smuc phosphorylation and ubiquitination. Both PAK5‐WT (Wild type construct) and PAK5‐S573N (activation construct) could increase Smuc expression, and PAK5‐S573N was more effective than PAK5‐WT (Figure 2A). However, there were distinct tendencies observed between PAK5‐K478M (Deactivation construct) and control (Figure 2A). All these suggested that PAK5 regulated the protein accumulation of Smuc. In vitro phosphorylation assays showed that PAK5‐S573N, but not PAK5‐K478M, strikingly stimulated the Ser278‐phosphorylation of Smuc (Figure 2B). PAK5‐S573N could markedly prolong the half‐life of Smuc protein (Figure 2C), and yet PAK5‐K478M could not effectuate the prolonged half‐life (Figure 2D). Smuc was more unstable in the pattern of Smuc‐S278A but not Smuc‐WT after PAK5 overexpression (Figure 2E). Furthermore, ubiquitination of Smuc was enhanced in PAK5‐K478M compared with PAK5‐WT (Figure 2F); however, it was scarcely detected in PAK5‐S573N (Figure 2F). Ubiquitination of Smuc was easily noticed in Smuc‐S278A than in Smuc‐WT after PAK5‐S573N overexpression (Figure 2G). These results suggested that PAK5‐mediated Smuc phosphorylation inhibited ubiquitination‐dependent Smuc degradation.

**FIGURE 2:**
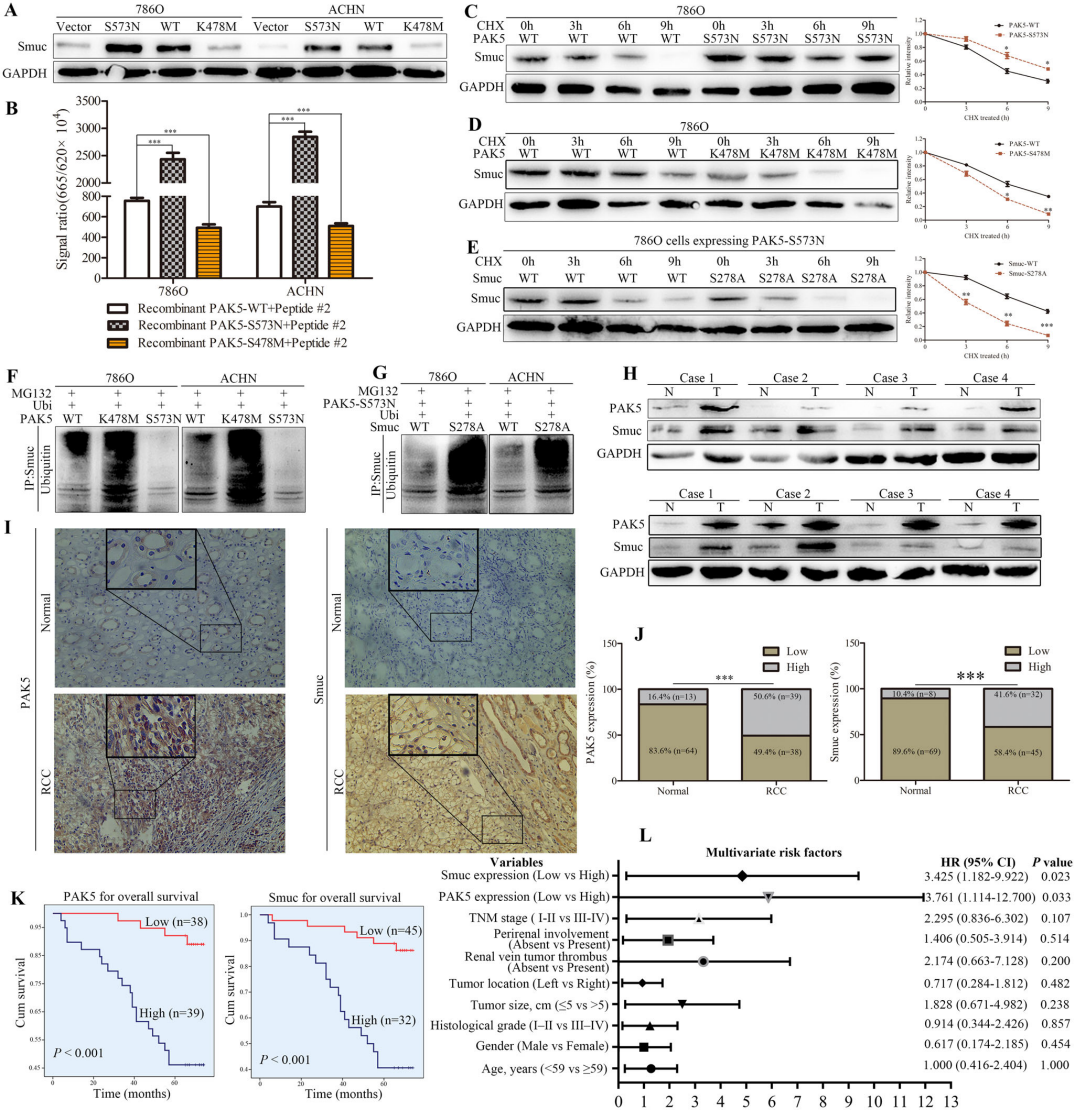
PAK5‐mediated Smuc phosphorylation suppresses its ubiquitination. (A) The effects of different PAK5 constructs (PAK5‐WT: wild type construct; PAK5‐S573N: activation construct; PAK5‐K478M: deactivation construct) on Smuc expression were detected in 786O and ACHN cells. (B) In vitro phosphorylation assay was employed to explore the effects of different PAK5 constructs on the phosphorylation level of Smuc/peptide #2 (potential PAK5‐phosphorylation site/Ser287 of Smuc). (C and D) Different constructs (PAK5‐WT, PAK5‐S573N and PAK5‐K478M) of PAK5 were transfected into 786O cells. Smuc protein expression was detected using western blot. (E) PAK5‐S573N was co‐transfected with Smuc‐WT or Smuc‐S278A in 786O cells. Smuc expression was detected after CHX treatment for the indicated time. (F) 786O and ACHN cells were treated with MG132, and PAK5‐WT/ S573N/K478M was respectively transfected into cells with Ubi plasmids. And ubiquitinated Smuc was immunobloted with an anti‐ubiquitin antibody. (G) PAK5‐S573N was co‐transfected with Smuc‐S278A or WT, and the ubiquitinated Smuc was detected. (H) RCC tissues (T) and matched normal tissues (N) were collected to explore PAK5 and Smuc expression. (I) Representative PAK5 and Smuc IHC staining in RCC tissues and adjacent tissues. (J) PAK5 and Smuc were increased in RCC tissues. (K) Kaplan‐Meier overall survival analysis of PAK5 and Smuc expression in RCC patients. (L) Multivariate regression analysis of potential predictive factors in RCC. *p < 0.05; **p < 0.01; ***p < 0.001; # > 0.05

PAK5 and Smuc in RCC tissues were highly expressed (Figure 2H). Strong staining of PAK5 and Smuc was frequently observed in RCC tissues using IHC, but sparse or negative staining was found in adjacent normal tissues (Figure 2I). In addition, 50.6% and 41.6% of RCC tissues presented increased PAK5 and Smuc expression (Figure 2J), respectively. Increased PAK5 was associated with histological grade (*p* = 0.029), gender (*p* = 0.027), and TNM stage (*p* = 0.009) (Table S1). Inconsistently, increased Smuc was correlated with TNM stage (*p* = 0.025) and perirenal involvement (*p* = 0.029) (Table S1). Simultaneously, PAK5 positively correlated with Smuc (Table S2, *R* = 0.411), which indicated that coordination between Smuc and PAK5 might elicit a positive action on RCC progression. Both PAK5 and Smuc predicted a shorter overall survival for RCC patients (Table 2K). And they may function as novel prognostic markers in RCC (Table S3; Figure 2L).

Cell proliferation was unaffected by either Smuc overexpression or knockdown (Figure S2A,B). Transwell and wound healing assays indicated that Smuc promoted cell migration and invasion in RCC (Figure S2C‐F). Smuc resulted in transforming the paving stone and sheet‐like RCC cells into fibroblast‐like spindle shape (Figure S2G). PAK5 accelerated the wound closure of RCC cells (Figure S3A). Noteworthy, silencing Smuc mitigated the migration, invasion, and tube formation activity induced by PAK5 overexpression (Figure 3A‐C; Figure S3B‐D). PAK5 remodeled cell morphology, and induced RCC cells extending the pseudopodium branching represented a more flexible migrating style by regulating Smuc (Figure S3E). Significantly, PAK5‐WT rather than K478M‐induced alterations of EMT markers were blunted with Smuc depletion (Figure 3D; Figure S4A). Immunofluorescence staining also showed that inhibiting Smuc rescued the E‐cadherin downregulation (Figure S4B). Smuc bound to E‐cadherin promoter and notably neutralized its transcription activity conferred by PAK5 silencing (Figure 3E‐G). Smuc blockade inhibited PAK5‐induced RCC metastasis in vivo (Figure 3H; Figure S4C). Furthermore, IHC results validated that PAK5 appreciably enhanced Smuc staining while it elicited a negative effect on E‐cadherin (Figure 3I); however, suppressing Smuc reversed staining alternations (Figure 3I). Consistently, PAK5‐induced EMT markers were abrogated after Smuc depletion in vivo metastatic models (Figure S4D). Additionally, IHC staining also confirmed the increased Smuc and PAK5 in metastatic RCC tissues (Figure 3J).

**FIGURE 3:**
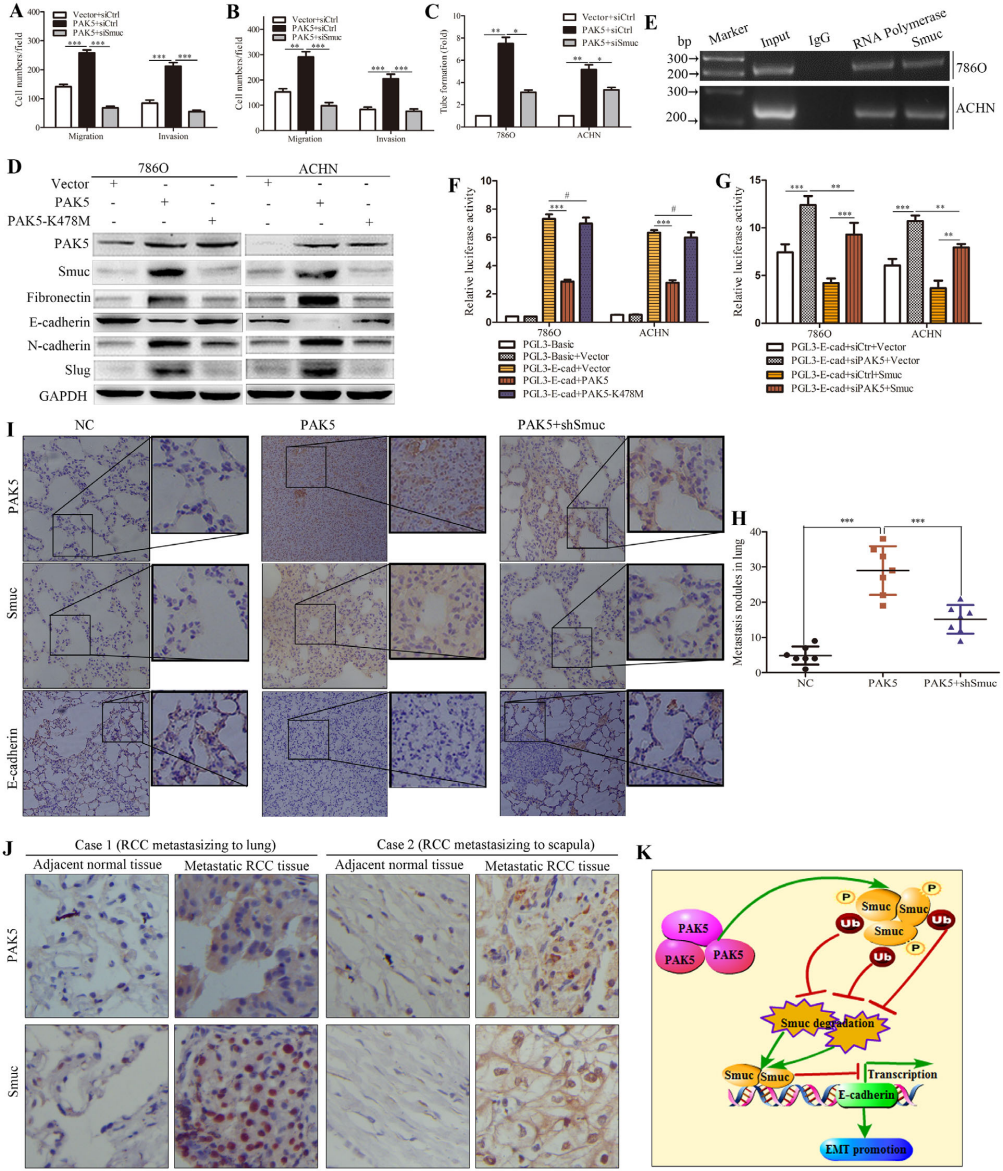
PAK5‐Smuc axis promotes the RCC metastasis by inducing EMT. (A and B) Transwell assays were performed to explore the effects of Smuc on PAK5‐induced cell migration and invasion. (C) The role of Smuc in PAK5‐induced tube‐like formation activity was explored by HUVEC tube formation assays. (D) The expression of EMT‐involved markers was examined using western blot after expressing PAK5‐WT and PAK5‐K478M in RCC cells. (E) ChIP‐PCR assay was utilized to determine the binding affinity of Smuc to E‐cadherin promoters in RCC cells. IgG and RNA polymerase functions as the negative and positive controls, respectively. (F) Transcription activity of E‐cadherin in PAK5‐WT/K478M was determined by luciferase reporter assays. (G) PAK5 knockdown increased the transcription of E‐cadherin, which was inhibited by Smuc in RCC cells. (H) In vivo metastatic models were established using 786O cells with lentivirus expression vectors. After 6 weeks of implantation, lung metastasis nodules were counted in NC, PAK5 and PAK5+shSmuc groups. (I) Sections of lung tissues from metastatic models were stained with PAK5, Smuc and E‐cadherin antibodies by IHC. (J) Representative IHC images of PAK5 and Smuc in metastatic RCC tissues and matched adjacent normal tissues were shown. (K) A model shows that PAK5‐mediated Smuc phosphorylation inhibits the ubiquitination‐dependent Smuc degradation, inducing EMT and cancer metastasis. *p < 0.05; **p < 0.01; ***p < 0.001; # > 0.05

In conclusion, we identify Smuc as a novel downstream partner of PAK5 and demonstrate that PAK5‐mediated Smuc phosphorylation impairs its ubiquitination degradation (Figure 3K). Mechanism dissections support the crucial potential for the PAK5‐Smuc axis in promoting the EMT and metastasis of RCC. Our study provides evidence of a de novo PAK5‐Smuc pathway in RCC progression and novel therapeutic targets for tumor metastasis.

## CONFLICT OF INTEREST

The authors declare that they have no competing interests.

## AUTHOR CONTRIBUTIONS

Fu‐Chun Huo, Zhi‐Man Zhu and Qiu‐Ying Du performed and analyzed experiments. Fu‐Chun Huo and Zhi‐Man Zhu wrote the paper. Qiu‐Ying Du analyzed and interpreted the data. Dong‐Sheng Pei obtained funding and designed the research.

## AVAILABILITY OF DATA AND MATERIALS

All data in our study are available upon request.

## ETHICS APPROVAL AND CONSENT TO PARTICIPATE

 The use of human subjects was approved by ethics committee of the Affiliated Hospital of Xuzhou Medical University, and written informed consent was obtained from the participants. All animal experiments were approved by the Institutional Animal Care and Use Committee of Xuzhou Medical University and in accordance with institutional guidelines.

## Supporting information



Supporting Information
