# Supplementary material for: PAK5‐stabilized Smuc confers renal cell carcinoma metastasis
Source: Clin Transl Med. 2021 Sep 16;11(9):e559. doi: 10.1002/ctm2.559 (PMC8444558; doi:10.1002/ctm2.559)
Supplement: Supplementary file 1 — Supporting Information [file CTM2-11-e559-s001.docx]

**SUPPORTING INFORMATION**

**PAK5 stabilized-Smuc confers renal cell carcinoma metastasis**

**The Authors:** Fu-Chun Huo^1,*^, Zhi-Man Zhu^2,*^, Qiu-Ying Du^1,*^, Dong-Sheng Pei^1,#^

**Affiliation:**

1. Department of Pathology, Xuzhou Medical University, 209 Tong-shan Road, Xuzhou 221004, Jiangsu, China

2. Department of Basic Medicine, Jiangsu College of Nursing, Huai’an, China

* Fu-Chun Huo, Zhi-Man Zhu and Qiu-Ying Du contributed equally to this study.

^#^ Corresponding authors:

Name: Dong-Sheng Pei

Address: Department of Pathology, Xuzhou Medical University, Xuzhou 221004, China

209 Tong-shan Road, Xuzhou, Jiangsu, China

Tel: +86-0516-85582513; E-mail: [dspei@xzhmu.edu.cn](mailto:dspei@xzhmu.edu.cn)

**Running Title:** PAK5-Smuc signaling in RCC.

**KEYWORDS:** PAK5, Smuc, metastasis, phosphorylation

**Abbreviations**

CCK-8, Cell Counting Kit-8; ChIP, chromatin immunoprecipitation; CHX, cycloheximide; EMT, epithelial-mesenchymal transition; IP, immunopurification, IHC, immunohistochemistry; IP, immunopurification; PAKs, p21-activated kinases; PAK5, p21-activated kinase 5; qPCR, quantitative real-time PCR; RCC, renal cell carcinoma;

**Supplemental Materials and methods**

**1 Cell culture**

The human RCC cell lines 786O and ACHN were purchased from the Cell Bank, China Academy of Science (Shanghai, China). ACHN cells were maintained in DMEM (Gibco, Grand Island, NY, USA). 786O cells were cultured in RPMI-1640 medium. Medium contains 10% FBS (Gibco) and 1% antibiotics. All cells were cultivated under the circumstance of containing 5% CO_2_ at 37 °C.

**2 Transfection**

Cells were transfected with siRNAs using silentfect Lipid Reagent (Bio-Rad, Hercules, CA, USA) according to the manufacturers’ protocol. The target sequences for siSmuc and negative control (siCtrl) were synthesized by GenePharma (Shanghai, China). siRNA sequences were as follows: siSmuc, 5’-CCUGCAAGUACUGCGACAA-3’; siPAK5, 5’-CAAAGTCTTCGTACCTGAATC-3’; siCtrl, 5’-UUCUCCGAACGUGUCACGUTT-3’. For generating stable cell lines, the lentiviral expression vector (GenePharma) for Smuc knockdown (LV-shSmuc), PAK5 overexpression (LV-PAK5) and negative control (LV-NC) was designed. The target sequence of shRNA was as follow: shSmuc, 5’-CCUGCAAGUACUGCGACAA-3’; negative control (shCtrl), 5’-UUCUCCGAACGUGUCACGUTT-3’. Infectious lentiviruses were selected with 2 μg/ml puromycin (Vicmed, Xuzhou, China)

**3 Western bolt and immunopurification (IP)**

Western bolt was performed as previously described.[^1^](#_ENREF_1) Cell samples were lysed with RIPA lysis buffer (Beyotime, Shanghai, China) mixed with 1% protease inhibitors. Proteins were separated by SDS-PAGE electrophoresis and transferred to nitrocellulose membranes, followed by incubating with the primary and corresponding HRP secondary antibodies. Finally, protein signals were detected by the enhanced chemiluminescence reaction. For the IP analysis, Smuc and negative control IgG antibodies were added to the corresponding supernatant for the incubation at 4 °C overnight. Protein A/G magnetic beads were employed to capture protein-protein complexes for the western bolt. Primary antibodies were as follows: Smuc (Santa Cruz, CA, USA), N-cadherin (abcam, MA, USA), E-cadherin (abcam), Fibronection (abcam), N-cadherin (abcam), Slug (Santa Cruz), Ub (Santa Cruz) and GAPDH (Zhongshan biotech, Beijing, China).

**4 Cell proliferation assay**

Cell proliferation ability was detected by Cell Counting Kit-8 (CCK-8; Vicmed). Transfected cells were inoculated into 96-well plate (4×10^3^ cells/well), and 10 ul of CCK-8 solution were added to each well with a 100 ul serum-free medium. After the incubation with the mixture for 1 h, the absorbance of 450 nm was detected using a Multi-function enzymelinked analyzer. Each experiment was duplicated three times.

**5 Transwell assay**

Cells mixed in the serum-free medium were incubated into the upper chambers (BD Bioscience, San Jose, CA, USA) with Matrige (for migration) or without Matrigel (for invasion) at 2×104 cells per chamber. There was a 600 uL medium with 10% FBS in the lower chambers. After the incubation for 24 h, cells were fixed with 4% paraformaldehyde and stained with haematoxylin. Each experiment was duplicated three times.

**6 Scratch assay**

Cells were seeded into 6-well plates. The cell layer was slowly scratched with a 200ul pipette tip when cells reached 90% confluence. After the incubation for indicated times, the wound healing was screened by a microscope. Each experiment was duplicated three times.

**7 Immunofluorescence staining**

Cells were seeded into 6-well plates with coverslips. Cells were fixed with 4% paraformaldehyde and permeabilized with 0.1% Triton X-100. Subsequently, Smuc antibody was incubated overnight at 4°C, followed by the visualization using Alexa Fluor 488/594-tagged secondary antibodies. And cell nuclei were stained using DAPI. The colocalization was scanned by a confocal scanning microscope.

**8 Protein turnover assay**

After transfection, cells were treated with 100 μg/mL of cycloheximide (CHX, a protein synthesis inhibitor) to inhibit global protein translation. Cells were harvested at the indicated time, and the expression of Smuc protein was measured by the Western bolt.

**9 Ubiquitination assay**

Ubiquitination assay was performed by the above IP protocol. Transfected cells were treated with 50 μg/mL of MG132 (a proteasome inhibitor) for 6 h, and cell lysates were immunoprecipitated with Smuc antibody. The protein complexes were probed with a Ub antibody to visualize the ubiquitinated Smuc proteins.

**10 Generation of the unphosphorylated and phosphorylated peptides**

Before the phosphorylation assay, we predicted a potential phosphorylation motif (Ser278) of Smuc mediated by PAK5 according to the principle and discipline of PAKs determining substrate phosphorylation.[^2^](#_ENREF_2) For identifying the specific site, 5 biotin-labeled peptides were generated. The residue sequences of peptides were as follow: peptide #1 (Containing the random disruption of the potential PAK5-phosphorylation site/Ser278 of Smuc and its around sequences): 269-LRARTCSKFTMHLRS-282; peptide #2 (Containing the potential PAK5-phosphorylation site/Ser287 of Smuc and its around sequences): 269-RCTKTFSRMSLLARH-282; peptide #3 (Meaning the single-site mutation of Ser278 to alanine of Smuc): 269-RCTKTFSRMALLARH-282; peptide #4 (Containing the identified PAK5-phosphorylation site/Ser39 of E47.^3^): 30-VTNGKGRPASLAGAQ-44; peptide #5: 30-VTNGKGRPAALAGAQ-44 (Meaning the single-site mutation of Ser39 to alanine of E47.[^3^](#_ENREF_3)).

**11 In vitro phosphorylation assay**

Phosphorylation assay was performed with homogeneous time resolved fluorescence (HTRF)^®^ KinEASE-STK (serine/threonine kinase) S2 kit (Cisbio, France) according to the manufacturer’s instruction. The KinEASE-STK S2 kit is suitable for the detection of serine/threonine kinase activities and contains identified phosphorylation substrate (STK substrate 2-biotin) by PAK5. In brief, we first prepared the reagents needed for the assay. The 5 × kinase buffer was diluted into 1 × kinase buffer containing DTT (1 mM; Sigma-Aldrich, USA) and MgCl_2_ (5 mM, Sigma). STK substrate 2-biotin was treated into a 500 mM solution with distilled water. And the synthetic peptides were diluted into 50 ul/L. Streptavidin-XL665 was diluted into 16.67 mM. Recombinant human PAK5 protein (Abcam, USA) was diluted into 1 ug/L and stored at -80 °C until use. Detection buffer was resuspended in distilled water and employed to prepare for the working solution of Streptavidin-XL665 and STK-antibody–cryptate. Adenosine triphosphate (ATP; Sigma) was diluted into 5 mM with 1 × kinase buffer.

The assay was performed with HTRF 96-well low volume plate (Cisbio). The 4 ul of 1 × kinase buffer, 2 µL of STK substrate 2-biotin or synthetic peptide solution, 2 µL of recombinant PAK5 protein solution and 2 µL of ATP were added in turn to 96-well. After sealing the plate and incubating for 2 min at room temperature, 5 µL of and STK antibody-cryptate (donor) and Streptavidin-XL665 (acceptor) respectively were added. After 1 h incubation, the acceptor and donor emission signals of each individual well were measured by a VICTOR Nivo multimode microplate reader (PerkinElmer, Waltham, MA, US). The ratio of acceptor and donor emission signals was calculated using 10^4^ × Signal 665 nm/ Signal 620 nm method.

**12 Quantitative real-time PCR (qPCR)**

Total RNA from cells was isolated using TRIzol reagent (Vicmed). And first-strandcomplementary DNA was synthesized using a One-Step RT-PCR Kit (TaKaRa, Japan) according to the manufacturer’s instructions. qRT-PCR analysis was performed using ABI StepOne Plus Real-Time PCR system with the SYBR Master Mixture (TaKaRa). The mRNA expression was analyzed using the 2^−ΔΔCt^ method. And GAPDH was used as a housekeeping gene. The primers for the qPCR were listed below: Smuc forward primer: 5’-GTGAAAACGCACTCCAGC-3’, reverse primer 5’-AGAGCAGGCACCATTGATT-3’; PAK5 forward primer: 5’-CCTCAGCCTCTCATCCAGCACC-3’, reverse primer: 5’-AGGGCCGCCCGAAACTGTT-3’; GAPDH forward primer: 5’-AGAAGGCTGGGGCTCATTT-3’, reverse primer: 5’-AGGGGCCATCCACAGTCTT-3’.

**13 Immunohistochemistry (IHC) staining**

A total of 77 pairs of RCC tissues and matched normal tissues were collected from the Affiliated Hospital of Xuzhou Medical University between 2013 and 2015. All patients underwent curative surgery without preoperative chemotherapy or radiotherapy and were pathologically confirmed RCC. Clinicopathologic information was stored completely. The study was approved by the ethics committee of the Affiliated Hospital of Xuzhou Medical University and performed after obtaining written informed consent from the participants.

IHC staining was performed as previously described.[^1^](#_ENREF_1) Briefly, after retrieving antigen, quenching peroxidase and blocking nonspecific staining, specimen slides were orderly incubated with the appropriate primary and secondary antibodies and then stained with DAB. Two independent pathologists evaluated the IHC staining of PAK5 and Smuc proteins. The immunoreactive score is a product of the staining intensity (0: -; 1: +; 2: ++; 3: +++) timing the positive cells (1: 0–25%); 2: 26–50%; 3: 51–75%); 4: 76–100%), ranging from 0 to 12 scores. According to the score, RCC cases were divided into low expression (score ≤ 4) and high expression (score > 4).

**14 HUVEC tube formation assay**

The 786O and ACHN cells were pretreated and the supernatant composed of the serum-free medium was collected. Matrigel (40 μl/well) was placed in a 96-well plate. After the incubation for 1 h at 37 °C, A total of 4x10^4^ were suspended with conditioned medium and seeded in an indicated 96-well plate. Tube-like formation of HUVECs was observed after the incubation for 5 h at 37 °C and photos were acquired by a microscope.

**15 F-actin phalloidin staining**

Cells were cultured on coverslips, fixed in 4% formalin and permeabilized with 0.5%

Triton X-100. The cells were then incubated with the phalloidin for 40 min. And the nuclei were stained with Hoechst 33342 for 5 min. A confocal image was taken under a fluorescence microscope.

**16 Dual luciferase reporter assay**

E-cadherin promoter was cloned into the pGL3-Basic luciferase vector. Appropriate luciferase vectors and pcDNA3.1 plasmids were co-transfected into cells. The luciferase activities were consecutively measured by a dual-luciferase reporter assay system (Promega, Madison, WI, USA). The relative Firefly/Renilla luciferase activities were calculated. Each experiment was duplicated three times.

**17 Chromatin immunoprecipitation (ChIP) assay**

ChIP assay on the E-cadherin promoter was performed with the EZ-ChIP kit (Millipore, Bedford, MA, USA) following the manufacturer’s instructions. Briefly, cells were fixed by 1% formaldehyde for 10 min. Subsequently, the lysates were treated with ultrasonic crushing to obtain chromatin DNA fragments and immunoprecipitated with anti-Smuc (Santa Cruz), negative control IgG and positive RNA pol II IgG antibodies for incubation overnight at 4 °C, which were then treated with protein A/G beads to capture the DNA-protein complexes. After been washed and eluted, precipitated DNA fragments of the promoter of Smuc were amplified and determined by PCR, using primers: forward, 5’-CAAGCTTCAATAGCCTGAGACAGGAGAGT-3’ and reverse 5’-GAAATTCGGCTGGAGTCTGAACTGACTT-3’.

**18 In vivo lung metastasis model**

The BALB/c female nude mice (4-week-old) were obtained by Beijing Huafukang (Beijing, China). A total of 18 nude mice was divided into 3 groups randomly. For the lung metastasis, stably expressing 786O cells (3 × 10^6^/0.1 mL) were injected into the tail vein of nude mice. After 7 weeks, all mice were sacrificed, and lung tissues were removed and stained by H&E and IHC staining. All animal experiments were approved by the Institutional Animal Care and Use Committee of Xuzhou Medical University and in accordance with institutional guidelines.

**19. Construction of PAK5 Knockout (KO)**

The CRISPR/Cas9 technology was employed to knock out the PAK5 gene (NCBI GeneID: 57144) in 786O cells as previously described.[^4^](#_ENREF_4) Briefly, small guide RNAs (sgRNAs) targeted PAK5 were designed by using the online gRNA design tool (http://chopchop.cbu.uib.no/). And two sgRNA sequence against PAK5 are as follows: GGGACATGAAAAGCCAGCACATC (sgRNA#1) and GGGTCCACCTAATGCTAGCTCGT (sgRNA#2). The sgRNAs were cloned into the lentiGuide-Puro plasmid (Addgene, Watertown, MA, USA). HeLa cells were transfected with the lentiGuide-Puro plasmid using Lipofectamine 2000 (Invitrogen). And puromycin was administered to select the positive cells after 48 h of infection. The efficiency of PAK5-KO in 786O cells was further verified by western blot.

**20 Statistical analysis**

Data are represented as the mean ± standard deviation (SD). Statistical analyses were performed by SPSS 19.0 or GraphPad Prism 5 software. The discrepancies between the two groups were assessed by Student t test, and multiple comparisons were analyzed by one-way ANOVA. Chi-square or Fisher’s exact test was applied to analyze the relationship between the expression of PAK5 and Smuc and the clinicopathological features of RCC patients. The Kaplan–Meier method with the log-rank test was used to perform survival curves. The correlation analysis between Smuc and PAK5 was evaluated by the Spearman correlation test. Univariate and multivariate Cox regression models were applied to explore the independent prognostic factors for overall survival. Values of *P* < 0.05 were considered statistically significant.

**References**

1. Huo FC, Pan YJ, Li TT, Mou J, Pei DS. PAK5 promotes the migration and invasion of cervical cancer cells by phosphorylating SATB1. Cell Death Differ. 2019;26(6):994-1006.

2. Tuazon PT, Spanos WC, Gump EL, Monnig CA, Traugh JA. Determinants for substrate phosphorylation by p21-activated protein kinase (gamma-PAK). Biochemistry. 1997;36(51):16059-16064.

3. Zhu G, Li X, Guo B, Ke Q, Dong M, Li F. PAK5-mediated E47 phosphorylation promotes epithelial-mesenchymal transition and metastasis of colon cancer. Oncogene. 2016;35(15):1943-1954.

4. Hu X, Peng WX, Zhou H, et al. IGF2BP2 regulates DANCR by serving as an N6-methyladenosine reader. Cell Death Differ. 2020;27(6):1782-1794.

**Supplemental Table**

| **Supplemental Table S1**. PAK5 and Smuc staining and clinicopathologic characteristics of RCC. | | | | | | | | | |
| --- | --- | --- | --- | --- | --- | --- | --- | --- | --- |
| Variables | Total | PAK5 staining (n = 77) | | |  | Smuc staining (n = 77) | | |  |
|  |  | Low (%) | High (%) | *P* value |  | Low (%) | High (%) | *P* value |  |
| Age |  |  |  | 0.910 |  |  |  | 0.576 |  |
| <59 years | 39 | 19 (48.7) | 20 (51.3) |  |  | 24 (61.5) | 15 (38.5) |  |  |
| ≥59 years | 38 | 19 (50.0) | 19 (50.0) |  |  | 21 (55.3) | 17 (44.7) |  |  |
| Gender |  |  |  | **0.027** |  |  |  | 0.419 |  |
| Male | 59 | 25 (42.4) | 34 (57.6) |  |  | 33 (55.9) | 26 (44.1) |  |  |
| Female | 18 | 13 (72.2) | 5 (27.8) |  |  | 12 (66.7) | 6 (33.3) |  |  |
| Histological grade |  |  |  | **0.029** |  |  |  | 0.061 |  |
| I–II | 41 | 25 (61.0) | 16 (39.0) |  |  | 28 (68.3) | 13 (31.7) |  |  |
| III–IV | 36 | 13 (36.1) | 23 (63.9) |  |  | 17 (47.2) | 19 (52.8) |  |  |
| Tumor size |  |  |  | 0.212 |  |  |  | 0.407 |  |
| ≤ 5 cm | 35 | 20 (57.1) | 15 (42.9) |  |  | 20 (57.1) | 15 (42.9) |  |  |
| > 5 cm | 42 | 18 (42.9) | 24 (57.1) |  |  | 25 (48.1) | 27 (51.9) |  |  |
| Tumor location |  |  |  | 0.420 |  |  |  | 0.656 |  |
| Left | 36 | 16 (44.4) | 20 (55.6) |  |  | 22 (61.1) | 14 (38.9) |  |  |
| Right | 41 | 22 (53.7) | 19 (46.3) |  |  | 23 (56.1) | 18 (43.9) |  |  |
| Renal vein tumor thrombus |  |  |  | **0.263*** |  |  |  | **0.265*** |  |
| Absent | 69 | 36 (52.2) | 33 (47.8) |  |  | 42 (60.9) | 27 (39.1) |  |  |
| Present | 8 | 2 (25.0) | 6 (75.0) |  |  | 3 (37.5) | 5 (62.5) |  |  |
| Perirenal involvement |  |  |  | **0.154*** |  |  |  | **0.029*** |  |
| Absent | 68 | 36 (52.9) | 32 (47.1) |  |  | 43 (63.2) | 25 (36.8) |  |  |
| Present | 9 | 2 (22.2) | 7 (77.8) |  |  | 2 (22.2) | 7 (77.8) |  |  |
| TNM stage |  |  |  | **0.009** |  |  |  | **0.025** |  |
| I–II | 52 | 31 (59.6) | 21 (40.4) |  |  | 36 (66.7) | 18 (33.3) |  |  |
| III–IV | 25 | 7 (28.0) | 18 (72.0) |  |  | 9 (39.1) | 14 (60.9) |  |  |
| Data were performed by Chi-square test; *Fisher’s Exact Test; Bold values show significance. | | | | | | | | | |

| **Supplemental Table S2**. The correlation of PAK5 and Smuc expression in RCC. | | | | | |
| --- | --- | --- | --- | --- | --- |
| Smuc | PAK5 | |  | Spearman | |
|  | Low | High |  | Rho | *P* value |
| Low | 30 | 15 |  | 0.411 | 0.000 |
| High | 8 | 24 |  |  |  |
| Total | 38 | 39 |  |  |  |

| **Supplemental Table S3**. Univariate Cox analysis of prognostic markers for OS in RCC patients. | | | |
| --- | --- | --- | --- |
| Prognostic variables | Univariate | | |
|  | HR | 95% CI | *P* value |
| Age, years (<59 vs ≥59) | 0.920 | 0.420-2.017 | 0.836 |
| Gender (Male vs Female) | 0.527 | 0.181-1.537 | 0.241 |
| Histological grade (I–II vs III–IV) | 1.702 | 0.772-3.752 | 0.187 |
| Tumor size, cm (≤5 vs >5) | 1.696 | 0.749-3.841 | 0.205 |
| Tumor location (Left vs Right) | 0.899 | 0.410-1.970 | 0.789 |
| Renal vein tumor thrombus (Absent vs Present) | 3.918 | 1.553-9.884 | **0.004** |
| Perirenal involvement (Absent vs Present) | 3.374 | 1.343-8.474 | **0.010** |
| TNM stage ( I-II vs III-IV) | 5.133 | 2.259-11.664 | ***P* < 0.001** |
| PAK5 expression (Low vs High) | 7.159 | 2.450-20.913 | ***P* < 0.001** |
| Smuc expression (Low vs High) | 6.224 | 2.475-15.655 | ***P* < 0.001** |
| HR, hazard ratio; 95% CI, 95% confdence interval; Bold values show significance. | | | |

**Supplemental Figures**


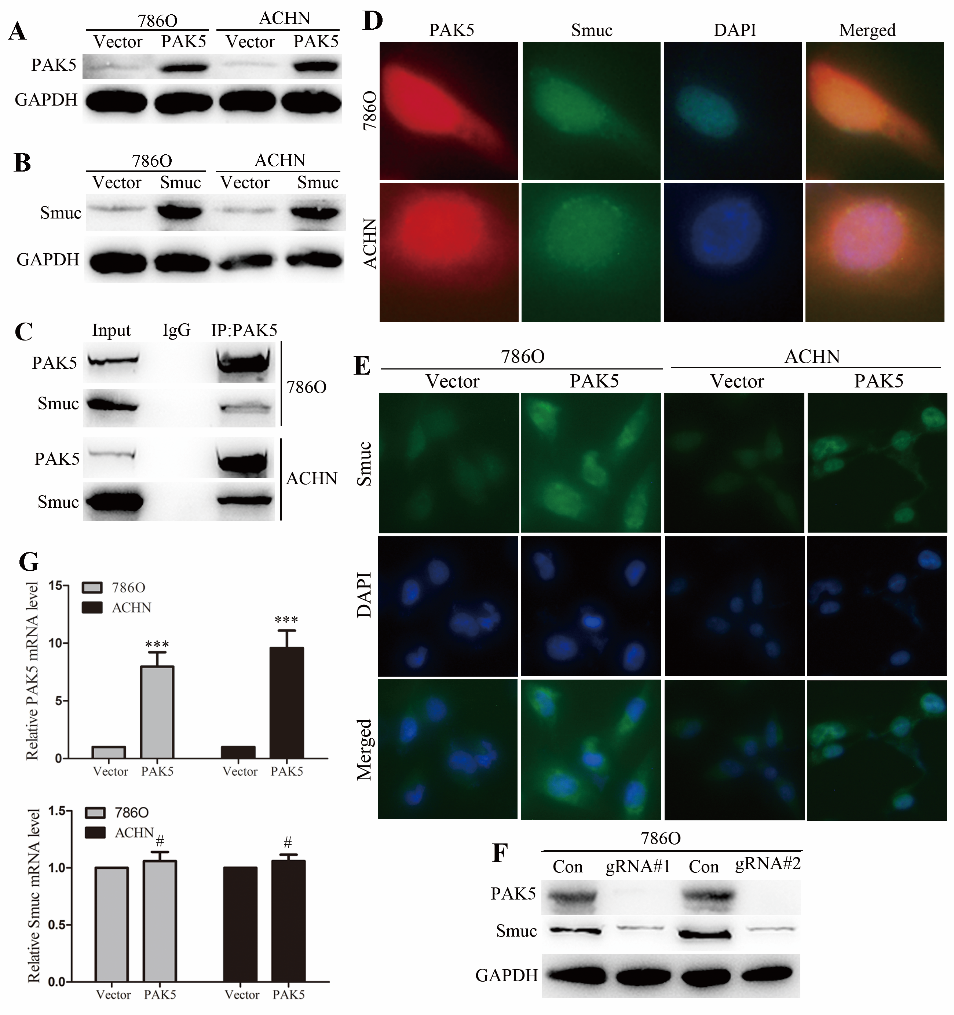


**Supplemental Figure S1** The effects of PAK5 on Smuc in RCC cells. A and B, Western blot was performed to confirm the overexpression of PAK5 and Smuc in RCC cells. C, Exogenous interaction between PAK5 and Smuc was determined by immunoprecipitation with the anti-PAK5 antibody in RCC cells. D, The co-localization of exogenous PAK5 and Smuc protein in RCC cells by immunofluorescence staining. E, Immunofluorescent staining of Smuc protein (green) in 786O and ACHN cells was performed in 786O and ACHN cells expressing PAK5. F, CRISPR/Cas9 technology was employed to knock out the PAK5 gene in 786O cells. And the effect of PAK5-KO on Smuc expression in 786O cells was further verified by western blot. G, The mRNA levels of PAK5 and Smuc was performed using qPCR in RCC cells expressing PAK5. ****P* < 0.001; # > 0.05.


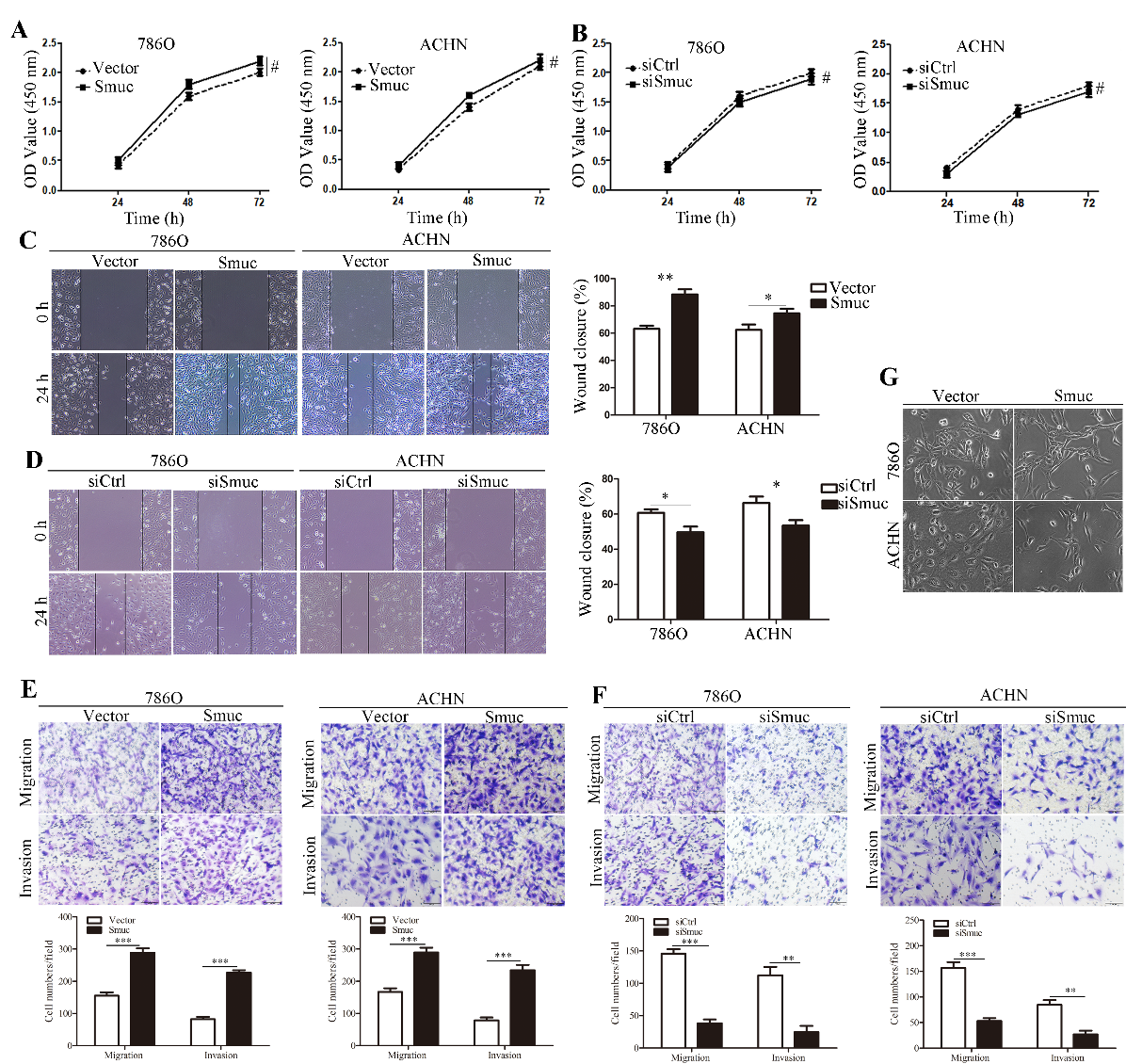


**Supplemental Figure S2** Smuc promotes the migration and invasion of RCC cells. A and B, CCK-8 proliferation assays were performed after transfecting Smuc plasmid or siRNA in 786O and ACHN cells. C and D, Wound healing was performed to examine the invasion ability of 786O and ACHN cells with indicated transfection. E and F, Transwell assays were performed to explore the effects of Smuc on migration and invasion of 786O and ACHN cells with Smuc overexpression and knockdown. G, Examining cell morphology under microscopy after cells with indicated transfections. **P* < 0.05; ***P* < 0.01; ****P* < 0.001; # > 0.05.


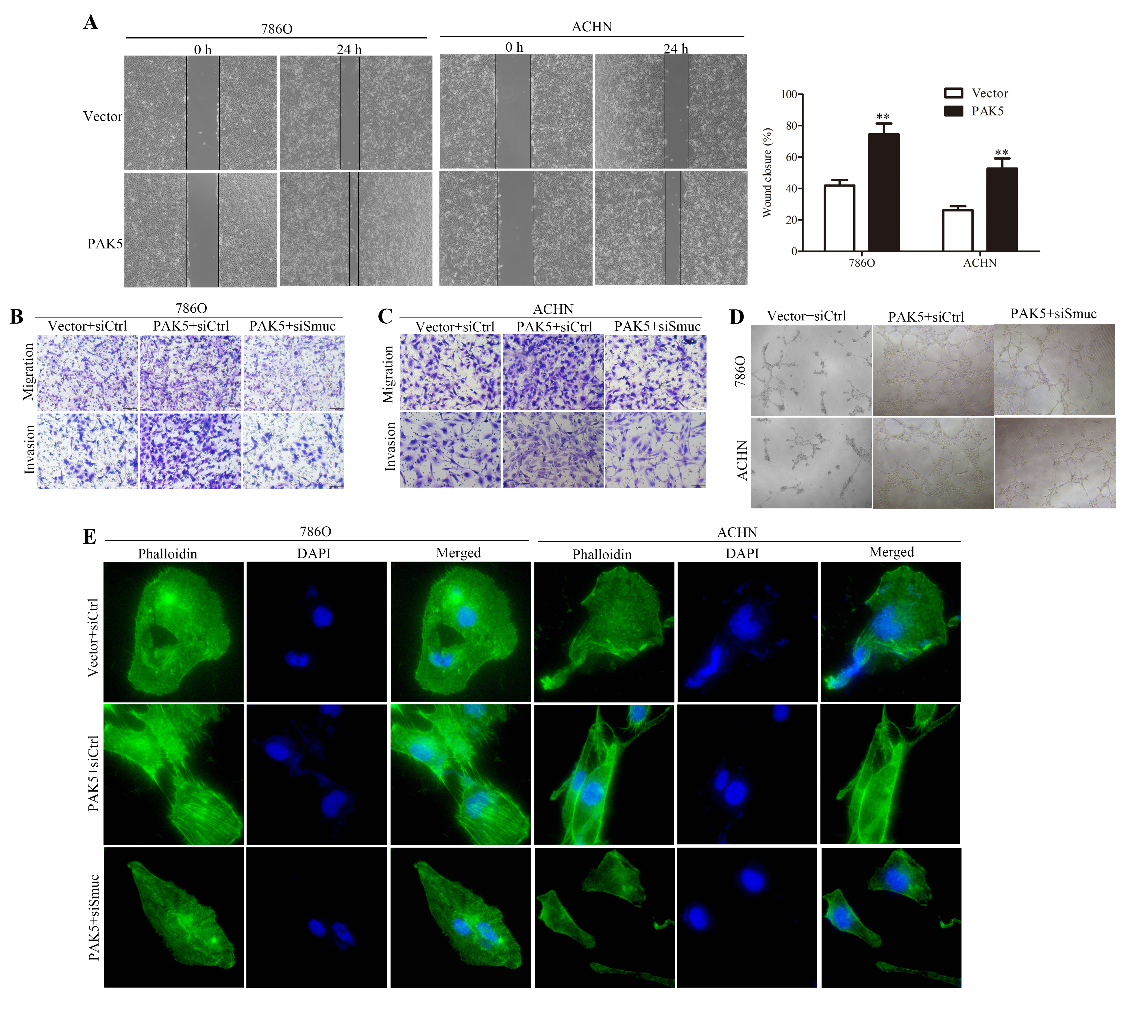


**Supplemental Figure S3** Smuc regulates PAK5-induced motility of RCC cells. A, The effect of PAK5 on the wound healing of RCC cells was investigated. B and C, Transwell assays were performed to explore the effects of Smuc on PAK5-induced migration and invasion of RCC cells. D, HUVEC tube formation assays were performed to explore the role of Smuc in PAK5-induced tube-like formation activity. E, F-actin phalloidin staining was performed to observe the cell morphology and the pseudopodium of cells after indicated treatment in RCC cells. ***P* < 0.01.


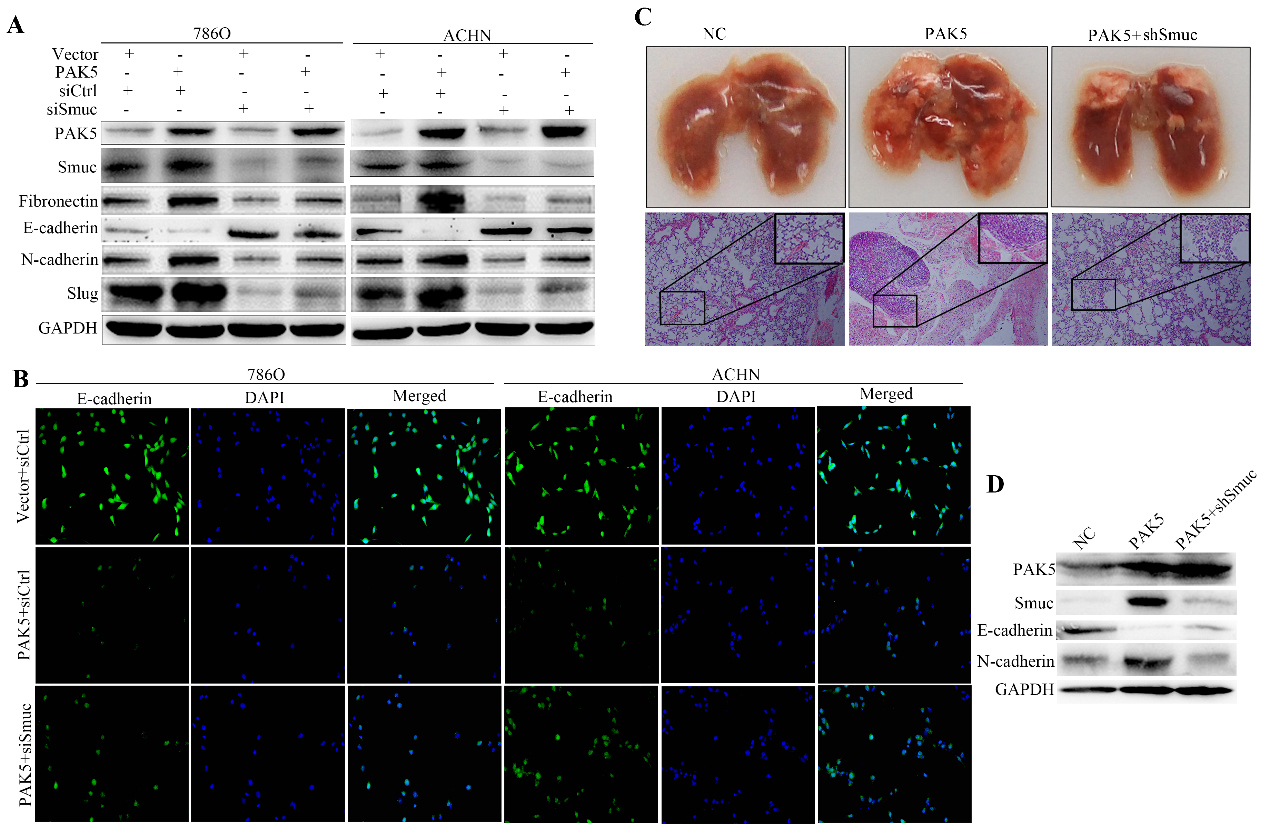


**Supplemental Figure S4** PAK5-Smuc axis promotes the RCC metastasis by inducing EMT. A, The effects of silencing Smuc on the alternations of PAK5-induced EMT markers were examined by western blot. B, Immunoﬂuorescent staining assay was performed to explore the role of Smuc in PAK5-induced E-cadherin in RCC cells. C, In vivo metastatic models were established using 786O cells with lentivirus expression vectors. After 6 weeks of implantation, lung metastasis nodules were counted in NC, PAK5 and PAK5+shSmuc groups. Lung metastatic lesions were confirmed using H&E staining. D. The effects of silencing Smuc on the alternations of PAK5-induced EMT markers in vivo metastatic models were examined by western blot.
